# Effect of Physical Activity Behaviors, Team Sports, and Sitting Time on Body Image and Exercise Dependence

**DOI:** 10.3390/sports12090260

**Published:** 2024-09-20

**Authors:** Emanuel Festino, Olga Papale, Francesca Di Rocco, Marianna De Maio, Cristina Cortis, Andrea Fusco

**Affiliations:** 1 Department of Human Sciences, Society and Health, University of Cassino and Lazio Meridionale, Viale dell’Università, 03043 Cassino, Italy; emanuel.festino@unicas.it (E.F.); olga.papale@unicas.it (O.P.); francesca.dirocco1@unicas.it (F.D.R.); marianna.demaio@unicas.it (M.D.M.); 2 Department of Medicine and Aging Sciences, University “G. d’Annunzio” of Chieti-Pescara, 66100 Chieti, Italy; andrea.fusco@unich.it

**Keywords:** body perception, exercise addiction, team sport, student athletes, sitting duration, volleyball, football, IPAQ

## Abstract

This study aimed to evaluate whether the type and degree of physical activity commitment (i.e., team sport athletes, highly active individuals, sedentary behavior) influence body image and exercise behavior. A total of 96 participants (46 women and 50 men; age: 22.7 ± 2.7 years; height: 170 ± 8.6 cm; weight: 67.6 ± 10.8 kg) were divided in six groups: female volleyball and male football players (student athletes classified as Tier 2), highly physically active women and men, with high sitting time completed the Body Image Dimensional Assessment and the 21-item Exercise Dependence Scale to evaluate body dissatisfaction and level of dependency on exercise. The Body Image Dimensional Assessment is a silhouette-based scale, where three direct indices are derived from the participants’ responses: body dissatisfaction, sexual body dissatisfaction, and comparative body dissatisfaction. The Exercise Dependence Scale is a six-point Likert scale that evaluates seven dimensions of exercise dependence (tolerance, withdrawal, intention effects, lack of control, time, reductions in other activities, and continuance) and provides an overall score. A multivariate analysis of variance was used to examine the effects of different levels of physical activity, team sport participation (volleyball and football), and sedentary behavior (i.e., high sitting time) on participants’ body image indices and dimensions of exercise dependence according to sex. Volleyball players showed significantly higher body dissatisfaction than football players (*d* = 0.99) and the physically active men group (*d* = 2.31), who had lower values than sedentary women group (*d* = 1.68). Football players had lower comparative body dissatisfaction values than volleyball players (*d* = 1.70) and sedentary women (*d* = 1.69), who had higher values than sedentary men (*d* = 1.04). Sedentary women had a significantly lower exercise dependence scale score than volleyball players (*d* = 1.71), football players (*d* = 1.12), and physically active men (*d* = 1.21). The findings highlight the impact of regular physical activity on body dissatisfaction and the variance in body image perceptions between the sexes. Moreover, the high exercise dependence values found in volleyball and football players and physically active men suggest an effect of intense training and maladaptive exercise behaviors, underlining the need for comprehensive strategies to address exercise dependence.

## 1. Introduction

The World Health Organization (WHO) defines mental health as a state of mental well-being that enables people to cope with the stresses of life, realize their goals, work productively, and contribute to their community [1]. This definition is reflected by the individual’s perception of their position in life, goals, expectations, standards, and concerns, defined as quality of life (QoL) [1]. Mental health is fundamental to well-being, and mental disorders are real health conditions, given their contribution to morbidity, premature mortality, and decreasing QoL [1]. To promote mental health, it is necessary to address negative health behaviors such as smoking, diet, alcohol consumption, and sedentary behaviors, such as long periods spent sitting [1,2,3].

Physical activity (PA) plays a key role in enhancing mental health. Engaging in regular PA, exercise (i.e., a subcategory of PA that is planned, structured, repetitive, and purposeful, aiming at the improvement in or maintenance of one or more components of physical fitness), or sports (i.e., a range of activities performed within a set of rules and undertaken as part of leisure or competition) [4] has been demonstrated to have a positive influence on mental health by reducing anxiety, depression, negative mood, and by improving confidence [5,6]. Exercise could also promote improvements in self-efficacy, as well as objective and perceived physical fitness (i.e., body composition, cardiorespiratory endurance, and muscular strength), all of which lead to improvements in body image [7,8]. Body image is a multidimensional construct that encompasses a behavioral aspect linked to body-related behaviors (e.g., checking behaviors), a perceptual aspect linked to the perception of body characteristics (e.g., estimation of one’s body size or weight), and a cognitive–affective aspect involving thoughts and feelings toward one’s body [7,8,9,10]. The contribution of exercise to improvements in body image might be attributed to the fact that individuals who engage in regular PA more closely resemble the aesthetic ideal of a lean and fit physique for women and a lean and muscular physique for men with respect to nonexercisers. The effect of exercise or sport on body image may also be due to the fact that PA participation is associated with an increase in psychological well-being that is related to a positive body image [11]. Negative thoughts, behaviors, perceptions, and feelings about one’s body are defined as body dissatisfaction (BD) [9,10,12], referring to the extent to which individuals are dissatisfied with their bodies, which has a significant impact on mental health. Sociocultural pressure regarding unrealistic ideals of beauty could lead to negative comparisons and a constant sense of inadequacy regarding one’s body, generating low self-esteem, anxiety, depression, and eating disorders [10,12,13,14]. This connection highlights the importance of addressing body image to foster mental health and prevent mental health issues and eating disorders.

In the sports and exercise context, positive body image is influenced by tangible physiological modifications, such as improvement in body composition, attributed to the sport practiced (individual vs. team sports), the individual’s perception of their physical changes, and the development of self-assurance and self-efficacy [8,15,16,17]. Although studies [8,15,16,17] have shown the benefits of sports on body image, athletes can experience BD due to the demands of their sport. In particular, some sports prioritize specific body types or unique attributes, and athletes may feel pressure to conform to these idealized body types, even if it means sacrificing their health or well-being. In individual sports, successful achievement depends on the success of the performance of the individual; so, some sports focusing on physical appearance and aesthetics (gymnastics, figure skating, etc.), and with certain weight classes (wrestling, boxing, etc.) could predispose the athlete to developing BD [18]. In team sports, performance-related tasks are shared among teammates, and success is determined by the individual achievement of each athlete [16], not only on their appearance. Furthermore, women tend to exhibit greater BD than their male counterparts [10,19]. Therefore, the role of sex in the relationship between body image and exercise or sport needs further study because the sex differences in the social ideals regarding one’s own body could have different influences the relationship between exercise or sport and body image [10,19].

BD may lead to exercise addiction, which can also result in decreased performance due to overload and physical burnout [8,15,20]. Despite studies showing how exercise and sport are beneficial for both psychological and physical health, there is a general consensus that excessive exercise may lead to addictive inclinations [21,22,23], such as dependence, characterized by a compulsion to train, resulting in uncontrollable excessive exercise behavior. This results in physiological and psychological symptoms that could have a negative impact on mental health, such as depression and anxiety [23]. Individuals who exercise frequently may feel unable to reduce their exercise; continue despite illness, injury, and social conflict; and experience “withdrawal symptoms” when forced to stop [23,24]. With BD, there are sex differences in exercise dependence, where men generally are at higher risk of developing maladaptive exercise behavior than women [25], given the greater motivation to exercise and increase the amount and intensity of training [25,26,27]. So, it is important to consider the role of sex in exercise dependence.

Because exercise has a positive effect on physical appearance, one of the reasons for exercise dependence is improving body image. Individuals exercise with the aim of enhancing fitness levels and achieving a desired physique, leading in turn to an increased risk of exercise dependence [28]. Athletes can also experience exercise addiction due to an obsession with achieving top performance and results [28]. Moreover, different sports elicit different physical, psychological, and social demands, all of which can significantly influence both self-perception and exercise-related behaviors [15]. Among the team sports, football and volleyball are the most popular within the Italian young adult population [29]. Therefore, this population could offer an accurate representation of the actual situation, providing useful information regarding body image and exercise dependence.

With sports being widely followed and practiced in Italy, football and volleyball players may experience strong television and social media exposure, influencing expectations and social pressures regarding body image and commitment to training, in particular in professional and college athletes [30,31,32]. Given the complex link between PA, perception of one’s own body, and excessive exercise [15,21,33], there is a need for an in-depth investigation that examines these dynamics in populations with different PA behaviors. Therefore, the aim of this study was to examine the effect of lifestyle, including team sport participation (i.e., football and volleyball), PA engagement level, and sitting time, and their effect on body image and exercise dependence according to sex. We hypothesized that team sport athletes, individuals with high levels of PA, and individuals with high sitting time significantly differ regarding BD, considering differences between the sexes, and that participants with high levels of PA and team sport athletes would show more exercise dependence than physically active individuals with long sitting time.

## 2. Materials and Methods

### 2.1. Study Design

In adherence to the Declaration of Helsinki, the research protocol, designed as a cross-sectional study, was approved on 8 March 2023 by the Institutional Review Board of the Department of Human Sciences, Society and Health at the University of Cassino and Lazio Meridionale (approval Number 9407) to examine the effects of team sport participation, PA engagement levels, and sitting time on BD and exercise dependence among university students. This cross-sectional study was conducted in the Sport and Exercise Physiology Laboratory of the University of Cassino and Lazio Meridionale, where all data were collected at a specific point in time, without any longitudinal follow-up. Data collection was conducted in the afternoon. The hour of data collection was chosen to accommodate the students’ and student athletes’ schedules, as they were free from training and university lectures at this time, ensuring their availability and minimizing conflicts with their academic and sport commitments. All participants provided informed consent and were informed of their right to withdraw from this study at any time and for any reason. The inclusion criteria were clearly defined to select student athletes who engaged in team sports, students regularly participating in PA, or with long sitting time. After the recruitment and selection process of the participants, the individuals’ body image and exercise dependence were evaluated.

### 2.2. Participants

Young adults were recruited among the student and student athlete population of the University of Cassino and Lazio Meridionale. To ensure that our study was robustly powered to detect substantial effects, we sought an effect size (ES) of 1.2, indicative of a very large impact by Cohen’s standards, to reflect our commitment to identifying only the most meaningful significant differences. The rationale behind targeting such a marked ES was from the existing literature [21], focusing on the relationship between PA and health perception. In particular, targeting an alpha of 0.05 and a power of 0.80 across 6 distinct groups, our power analysis demonstrated that a sample size of approximately 16 participants per group was required for obtaining an ES of 1.2. As highlighted by Schweizer and Furley [34], a large ES was chosen because smaller samples have a higher likelihood of producing type II error (i.e., not yielding a significant test although the effect exists). Moreover, small samples make replication attempts particularly difficult. This may mean that the effect does not exist or that the study did not have enough power to detect it. For these reasons, a power of 0.80 and a large ES (1.2) was chosen. A convenience sampling method was used to recruit participants from the university population by means of flyers, posters, brochures, and advertisements on social network. To avoid the influence of age in our evaluation, only participants in the young adult category (aged between 18 and 35 years) were recruited. After the recruitment and selection processes, different groups were divided based on their athletic and nonathletic status and sex: male football players and female volleyball players, highly physically active men and women, and men and women with long sitting time.

### 2.3. Measures

The Italian short version (7 items) of the International Physical Activity Questionnaire (IPAQ) was administered to the participants to evaluate the individual PA levels and sitting time. The IPAQ (Cronbach’s α coefficient of 0.60 [35]) comprises 7 items that evaluate the frequency, intensity, and duration of PA at various levels: low (such as walking), moderate, and vigorous, along with total PA per week. Additionally, it includes an item regarding daily sitting time to estimate sedentary lifestyle patterns. The IPAQ includes both categorical and continuous scores. The categorical scores classify participants into three levels: inactive, minimally active, and health-enhancing physical activity (HEPA) active, which denotes activity levels that exceed the minimum public health PA recommendations, which are associated with enhanced health benefits [35,36]. The continuous scores are calculated in metabolic equivalent of task (MET) minutes per week.

To classify people according to the sports practiced, the participants were asked: “Do you currently have any injuries that prevent you from training or exercise? Do you practice any sports? If yes, please specify which sport and for how long.” In fact, engaging in structured and regular training and competition exceeding the suggested [37,38] minimum amount of PA to obtain health benefits (i.e., exercising for more than 300 min per week at moderate intensity or 150 min per week exercises at vigorous intensity) could be beneficial for improving mental health among adults. Team sports in particular may provide even more powerful and additional benefits [39] due to the required intermittent physical exercise, high cognitive–attention demands, and problem-solving skills under time pressure [40]. Since sedentary behavior increases the risks of heart and metabolic diseases and the prevalence of psychological distress in adults, independent of the protective effect of leisure-time PA [41], it is crucial to consider the impact of long sitting time (>5 h per day) on mental and physical health [42,43,44]. Therefore, sitting time was used as indicator of sedentary behavior [45].

### 2.4. Procedures

**Figure 1:**
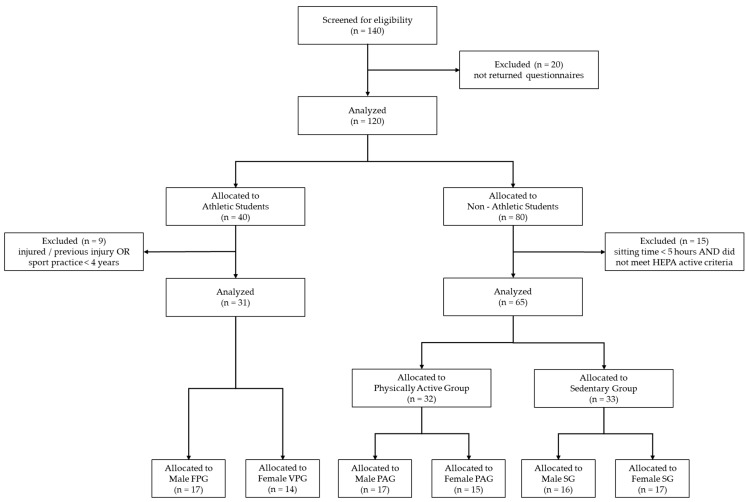
Flow chart of the recruitment and selection process of the participants included in the study. FPG = football players’ group, VPG = volleyball players’ group; PAG = physically active group; SG = sedentary group, HEPA = health-enhancing physical activity.

From a total of 140 participants (Figure 1), 20 were excluded because they did not fully complete the questionnaires. The remaining 120 participants were allocated according to their athletic status. If the participants reported practicing sports, they were allocated to the athletic students group, and the following inclusion criteria were applied: (i) student athletes engaged in the most popular Italian sports for their respective sexes [29], being football for men and volleyball for women; (ii) team sport training and competition for at least the previous 4 years. Participants were excluded if they had an injury that prevented them from training and competition or they practiced team sport for less than 4 years. From the 40 participants allocated to the athletic students’ group, 9 were excluded because of not meeting the inclusion criteria, while 31 were allocated to the football players’ group (*n* = 17) and volleyball players’ group (*n* = 14). These inclusion and exclusion criteria were chosen to ensure that the student athletes had substantial and consistent experience in their respective sports to avoid the impact of variables such as injury or insufficient training duration on the evaluation. Participants included in the football and volleyball players’ groups reported training on average for three 2 h sessions plus a competitive match per week. In according with the 6-tiered Participant Classification Framework [46], football and volleyball players were classified as Tier 2, corresponding to individuals engaging in sport-specific training approximately three times a week with the intention to compete at the local level.

If participants did not report practicing sport, they were allocated to the nonathletic students and based on the IPAQ responses; the following inclusion criteria were applied: (i) students classified as HEPA active, or (ii) students that reported sitting for 5 or more hours in total per day. Nonathletic students were excluded if they reported less than 5 h per day of sitting time while not meeting the criteria to be classified as HEPA active. From the 80 participants allocated to the nonathletic students group, 15 were excluded because of not meeting the inclusion criteria. Therefore, the 65 participants from the nonathletic students group were included and allocated to the physically active group (physically active men group: *n* = 17; physically active women group: *n* = 15) and sedentary group (sedentary men group: *n* = 16; sedentary women group: *n* = 17). The physically active group included participants who met one of two criteria to be classified as HEPA active: either engaging in vigorous-intensity activity on at least 3 days per week, achieving a minimum of 1500 MET minutes per week; or participating in any combination of walking, moderate-intensity, or vigorous-intensity activities totaling at least 3000 MET minutes per week [35,36]. The sedentary group included individuals who reported sitting for 5 or more hours in total each day. The inclusion of participants in the sedentary group was independent of their actual levels of PA, whether inactive, minimally active, or HEPA active. This approach acknowledges that even individuals who engage in regular PA can still lead a largely sedentary lifestyle. The categorization in the sedentary group was based solely on the amount of time spent sitting, reflecting a lifestyle with minimal physical movement or exertion. The long sitting time in this group corresponds to an energy expenditure ranging from 1.0 to 1.5 METs, which is characteristic of sedentary behavior [36,44,47]. The threshold of 5 or more hours of sitting per day was selected based on the literature [44,45,47] that has identifies this time as the critical point having impacts on health outcomes such as increased risks of mortality, metabolic syndrome, and cardiovascular diseases and having negative psychological effects.

Before starting the experimental session, participants’ anthropometric characteristics were collected. Body mass (kg) and height (m) measurements were recorded using a Seca 709 scale equipped with an integrated stadiometer, with precision up to 0.1 kg for weight and 0.1 cm for height (Vogel & Halke, Hamburg, Germany). Body mass index (BMI) was calculated using the formula of weight in kilograms (kg) divided by the square of height in meters (m^2^). All participants were classified as young adults (aged between 18 and 35 years) [48] and had a BMI within the normal range (18.5–24.9 kg/m^2^). The characteristics of the participants are presented in Table 1.

**Figure 2:**
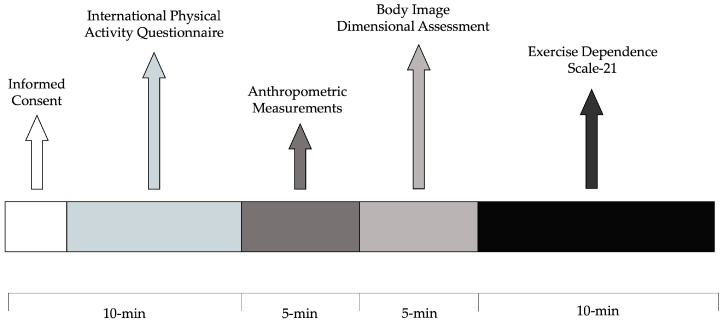
Timeline of the experimental procedures.

The data collection (around 30 min) was individually carried out under supervision of doctoral and trainee masters students in preventive and adaptive physical activity of the University of Cassino and Lazio Meridionale. Participants completed the Body Image Dimensional Assessment (BIDA) instrument and the 21-item Exercise Dependence Scale (EDS-21), described in detail in Section 2.5. The timeline of the procedures is shown in Figure 2.

### 2.5. Instruments

#### 2.5.1. Body Image Dimensional Assessment

The BIDA instrument adapted to Italian [21,49] was used to evaluate participants’ BD in relation to their body size. The BIDA was designed to measure the subjective and emotional dimensions of body image through a neutral, silhouette-based scale that is not specific to sex or ethnicity. The silhouette-based scale approach was chosen due to its effectiveness in minimizing biases from detailed and/or realistic images, focusing instead on basic body shape perceptions. Participants were asked to select silhouettes that represented their perceived and ideal body shape, the body shape they believe is most prevalent among their peers, and the body shape they perceive as most attractive to the opposite sex. The scale offered a range of figures depicting different body shapes, extending from 1.8 to 5.2. Participants were not confined to selecting only the numerical values corresponding directly to images on the scale: they could also choose intermediate values for which representative images were not provided. Three direct indices were derived from the participants’ responses:

- BD: This index represents a discrepancy between the participant’s actual and ideal body image.
- Sexual body dissatisfaction (SxBD): This index indicates the difference between the participant’s current body image and the body shape perceived as most attractive to the opposite sex.
- Comparative body dissatisfaction (CBD): This index measures the difference between the participant’s current body image and the perceived body image of the majority of peers of the same sex and age.

These indices are computed based on the numerical differences between the chosen silhouettes, providing quantitative measures of body dissatisfaction dimensions. The primary objective of the BIDA is to determine the degree to which a participant’s body image aligns with their desired body image. The three indices are expressed as percentages, ranging from −100% to +100%. Positive values indicate that the participant’s actual rating is higher than desired, than what is perceived as sexually attractive, or than the average among peers. Conversely, negative values suggest a lower self-assessment. Furthermore, a composite score, the Body Dissatisfaction Index (BDI), was computed as an indirect measure. This index is the mean of the absolute values of BD, SxBD, and CBD, ranging from 0 to 100%. A BDI score exceeding 30% is considered indicative of a potential risk of body image disorders. The BIDA showed good reliability (standardized Cronbach’s α coefficient = 0.881) in the nonclinical sample [49].

#### 2.5.2. Exercise Dependence Scale-21

The adaptation of the 21-item Exercise Dependence Scale (EDS-21) to Italian was used to assess the level of dependency on exercise among the participants [21,50]. This questionnaire uses a 6-point Likert scale, where 1 indicates ‘never’ and 6 ‘always’, to rate participants’ exercise behaviors. The EDS-21 focuses on seven key aspects to determine the potential addiction to exercise:

1. Withdrawal effects: This involves recognizing signs such as anxiety or fatigue that are typical when exercise is not performed or the need to maintain exercise intensity to avoid these symptoms.
2. Continuance: This is the tendency to sustain exercise routines even in the face of ongoing psychological or physical problems, such as injuries.
3. Tolerance: This reflects the requirement to progressively exercise more to achieve the same level of satisfaction or effect.
4. Lack of control: This involves challenges faced in attempting to reduce or regulate the amount of exercise volume and/or intensity.
5. Reduction in other activities: This is the inclination to limit social, work-related, or recreational activities in favor of exercise.
6. Time: This is when a considerable amount of time is spent in preparing for, engaging in, or recovering from exercise.
7. Intention effects: This is regularly performing more exercise than initially planned.

The identification of exercise dependence risk is based on achieving scores >14 in at least three of these seven dimensions. The overall score on the EDS-21 was computed by adding up the responses to all 21 questions [51]. The EDS-21 showed good psychometric characteristics (Cronbach’s α coefficients: withdrawal effects = 0.79; continuance = 0.74, tolerance = 0.87; lack of control = 0.87; reduction in other activities = 0.70; time = 0.85 and intention effects = 0.89) [50].

### 2.6. Statistical Analysis

STATA software version 14.2 (StataCorp, College Station, TX, USA) was used for statistical analysis. The Shapiro–Wilk test was employed to assess the normal distribution of the data. Means, standard deviations (SDs), and 95% confidence intervals (95% CIs) for continuous variables and frequencies and percentages for categorical variables were calculated. Internal consistency reliability of BIDA and EDS-21 was tested using Cronbach’s α coefficient.

One-way ANOVAs were used to examine IPAQ, BDI, and EDS score differences between PA groups (football players’ group, volleyball players’ group, physically active men, physically active women, sedentary men, and sedentary women). A multivariate analysis of variance (MANOVA) was used to examine the effects of different PA groups on participants’ body image indices (BD, SxBD, CBD) and exercise dependence (tolerance, withdrawal, intention effects, lack of control, time, reductions in other activities, continuance), separately.

The ES was calculated and is expressed as Cohen’s *d* and eta squared (η^2^) to determine the magnitude of the effects. The following criteria were used for the interpretation of Cohen’s *d*: small = 0.20, medium = 0.50, and large = 0.80. The thresholds for considering effects as small, medium, or large were values of η^2^ of 0.01, 0.06, and 0.14. For all the analyses, when significant main effects (*p* < 0.05) were found, Bonferroni correction was applied with a resulting *p*-value set at 0.003, and subsequently unpaired *t*-tests were performed across groups.

## 3. Results

The BIDA showed good internal consistency reliability, with a Cronbach’s α coefficient = 0.72 for the test scale based on all items. For single items, Cronbach’s α was 0.66 for BD, 0.53 for SxBD, and 0.70 for CBD. The EDS-21 showed an excellent reliability, with a Cronbach’s α = 0.84 for the test scale based on all items. For single items, Cronbach’s α was 0.83 for withdrawal effects, 0.85 for continuance, 0.82 for tolerance, 0.80 for lack of control, 0.82 for reduction in other activities, 0.81 for time and 0.82 for intention effects.

Based on the IPAQ score, all groups were minimally active (at least 150 min of moderate-intensity exercise or at least 60 min of vigorous-intensity exercise or 600 total METs per week) achieving the HEPA active category, except for the sedentary women’s group [36]. The results of the one-way ANOVA showed significant differences among the groups across several variables of the IPAQ scores (Table 2), including sitting time (F_(5, 90)_ = 28.77; *p* < 0.0001; η^2^ = 0.65), vigorous METs (F_(5, 90)_ = 8.80; *p* < 0.0001; η^2^ = 0.32), moderate MET (F_(5, 90)_ = 4.16; *p* = 0.0019; η^2^ = 0.18), and total METs (F_(5, 90)_ = 6.56; *p* < 0.0001; η^2^ = 0.26). Post hoc analyses with Bonferroni adjustments indicated that sedentary men and sedentary women exhibited significantly longer sitting time (*p* < 0.001 for both) compared to the other groups (football players’ group, volleyball players’ group, physically active men, physically active women). In terms of vigorous METs and total METs, both the physically active men and physically active women demonstrated significant differences from the other groups (football players’ group, sedentary men, sedentary women), with *p*-values of less than 0.003 and 0.001, respectively.

Regarding the participants’ body image (Table 3), the MANOVA results revealed a significant multivariate effect of PA level on the combined body image indices (BD, SxBD, CBD) (Wilks’ Lambda = 0.5726; F_(15, 243.3)_ = 3.63; *p* < 0.0001; η^2^ = 0.17; 95% CI = 0.02 to 0.27). Follow-up one-way ANOVA indicated significant effects of PA level on BD (F_(5, 90)_ = 5.16; *p* = 0.0003; η^2^ = 0.22) and CBD (F_(5, 90)_ = 6.39; *p* < 0.0001; η^2^ = 0.26). Subsequent to Bonferroni adjustments for multiple comparisons, significant differences emerged in the BD between the football players’ group and volleyball players’ group (*p* = 0.001; 95% CI = 6.27 to 24.07; t = 3.39; SE = 4.48; *d* = 0.99), between the physically active men and volleyball players (*p* < 0.001; 95% CI = −28.74 to −10.94; t = −4.43; SE = 4.48; *d* = 2.31), and between the physically active men and sedentary women (*p* = 0.001; 95% CI = −23.51 to −6.59; t = −3.53; SE = 4.25; *d* = 1.68).

**Figure 3:**
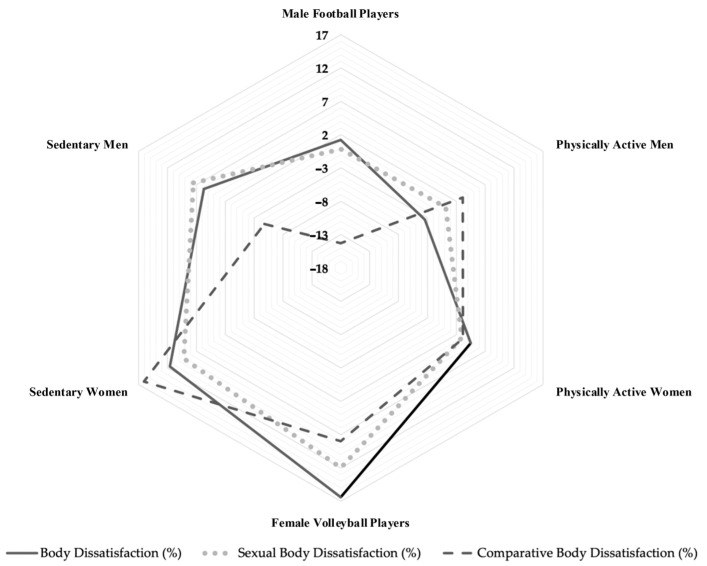
Radar chart of Body Image Dimensional Assessment indices, body dissatisfaction, sexual body dissatisfaction, and comparative body dissatisfaction, of team sport athletes, physically active participants, and participants with a predominantly sedentary lifestyle. Solid, dotted and dashed lines represent body dissatisfaction, sexual body dissatisfaction and comparative body dissatisfaction, respectively.

In terms of CBD, after accounting for multiple comparisons, the football players’ group showed significant differences when compared to both the volleyball players’ group (*p* < 0.001; 95% CI = 10.11 to 34.39; t = 3.64; SE = 6.11; *d* = 1.70) and the sedentary women (*p* < 0.001; 95% CI = 18.84 to 41.91; t = 5.23; SE = 5.80; *d* = 1.69). For the sedentary groups, a significant difference in CBD was found between the sedentary women and sedentary men, with a *p*-value of 0.001 (95% CI = −32.60 to −9.17; t = −3.54; SE = 5.89; *d* = 1.04). A graphical representation of the means of the body image indices among the groups is presented in Figure 3.

The one-way ANOVA indicated that there were no statistically significant differences in the BDI scores among the different PA groups (F_(5, 90)_ = 1.55; *p* = 0.1816; η^2^ = 0.079). Moreover, no group had a BDI score > 30%, which is the threshold value indicative of individuals at risk of body image disorders [49].

The means and standard deviations of the EDS-21 dimensions and the EDS score are presented in Table 4.

**Figure 4:**
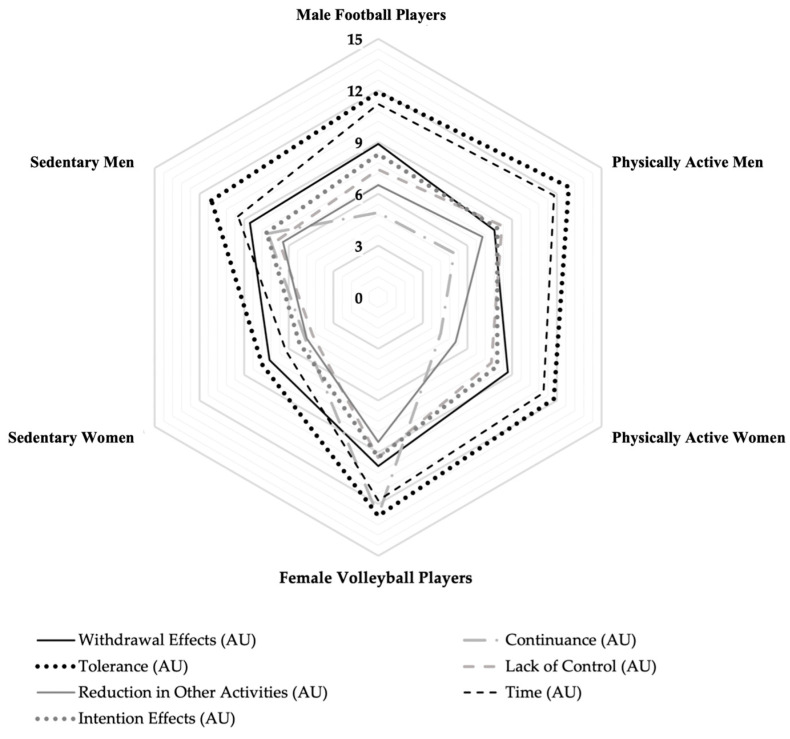
Radar chart of seven dimensions of 21-item Exercise Dependence Scale: tolerance, withdrawal effects, intention effects, lack of control, time, reductions in other activities, and continuance for athletes engaged in team sports, physically active participants, and participants with a predominantly sedentary lifestyle. Solid black, dotted black, solid grey, dotted grey, dotted and dashed, dashed grey, and dashed black lines represent withdrawal effects, tolerance, reductions in other activities, intention effects, continuance, lack of control, and time, respectively.

The MANOVA results revealed a significant multivariate effect of PA level on exercise dependence (tolerance, withdrawal, intention effects, lack of control, time, reductions in other activities, continuance) (Wilks’ Lambda = 0.3112; F_(35, 355.8)_ = 3.25; *p* < 0.0001; η^2^ = 0.15; 95% CI = 0.01 to 0.25). Follow-up ne-way ANOVA indicated significant effects of PA level in several aspects: tolerance (F_(5, 90)_ = 3.66; *p* = 0.0046; η^2^ = 0.16), lack of control (F_(5, 90)_ = 4.16; *p* = 0.0019; η^2^ = 0.18), time (F_(5, 90)_ = 7.05; *p* < 0.0001; η^2^ = 0.28), reductions in other activities (F_(5, 90)_ = 3.89; *p* = 0.003; η^2^ = 0.17), and continuance (F_(5, 90)_ = 13.94; *p* < 0.0001; η^2^ = 0.43). After adjustments using Bonferroni correction, significant differences were found. The sedentary women scored significantly lower than the volleyball players in several categories: lack of control (*p* < 0.001; 95% CI = −7.38 to −2.61; t = −4.17; SE = 1.20; *d* = 1.49), time (*p* < 0.001; 95% CI = −8.07 to −3.28; t = −4.71; SE = 1.20; *d* = 1.56), reductions in other activities (*p* < 0.001; 95% CI = −5.52 to −1.80; t = −3.92; SE = 0.93; *d* = 1.07), tolerance (*p* = 0.001; 95% CI = −7.71 to −2.06; t = −3.44; SE = 1.42; *d* = 1.15), and continuance (*p* < 0.001; 95% CI = −10.18 to −5.47; t = −6.61; SE = 1.18; *d* = 1.99). Additionally, the volleyball players’ group showed significantly higher values than the football players’ group in continuance (*p* < 0.001; 95% CI = 5.41 to 10.12; t = 6.56; SE = 1.18; *d* = 1.79) as well as compared to the sedentary men, physically active men, and physically active women in the same category, and in reduction in other activities than physically active women (*p* = 0.001; 95% CI = −5.14 to −1.31; t = −3.35; SE = 0.96; *d* = 0.94). Significant differences were also observed in the sedentary women from the other groups in terms of time. Specifically, the sedentary women differed significantly from the football players’ group (*p* < 0.001; 95% CI = −7.27 to −2.72; t = −4.36; SE = 1.14; *d* = 1.53) and from the physically active women (*p* < 0.001; 95% CI = 2.53 to 7.24; t = 4.13; SE = 1.18; *d* = 1.41). Additionally, the sedentary women showed significantly lower values than the physically active men in time (*p* < 0.001; 95% CI = 3.31 to 7.86; t = 4.88; SE = 1.14; *d* = 1.73), tolerance (*p* < 0.001; 95% CI = 2.31 to 7.68; t = 3.70; SE = 1.34; *d* = 1.32), and lack of control (*p* = 0.001; 95% CI = 1.67 to 6.20; t = 3.45; SE = 1.14; *d* = 1.30). A graphical representation of the means of the EDS-21 dimensions and the EDS scores among the groups is presented in Figure 4. Moreover, no group reached < 14 points on three of the seven dimensions; therefore, they did not present a significant risk of exercise dependence [51].

The one-way ANOVA showed significant differences in the EDS scores among the different PA groups (F_(5, 90)_ = 6.39, *p* < 0.0001, η^2^ = 0.26). The subsequent post hoc analysis indicated that the sedentary women had significantly lower EDS scores than the volleyball players’ group (*p* < 0.001; 95% CI = −45.66 to −21.58; t = −5.55; SE = 6.06; *d* = 1.71), the football players’ group (*p* = 0.002; 95% CI = −30.15 to −7.25; t = −3.25; SE = 5.76; *d* = 1.12), and the physically active men (*p* = 0.001; 95% CI = 8.67 to 31.56.; t = 3.49; SE = 5.76; *d* = 1.21).

## 4. Discussion

This study examined the effects of lifestyle, including team sport participation, PA levels, and sedentary behavior on body image and exercise dependence. Regarding body image, the findings suggested that sex and the level of PA significantly affected participants’ BD across the measured indices. A total of 42.74% of the variance in the BD indices could be attributed to in sex and PA level differences among the groups, highlighting the substantial effect of PA and sex differences on body image perceptions. In particular, differences in BD were observed between the football players’ group and volleyball players’ group, as well as between the physically active men and both the volleyball players and sedentary women, indicating that the type of sport, the level of PA, and sex play a role in influencing body image perceptions. In the CBD, differences were found between football players and both volleyball players and sedentary women, and between the sedentary groups, with women showing different from the sedentary men, highlighting meaningful differences in body image perceptions across groups with different PA levels. PA emerged as also having an impact on exercise dependence, with 68.88% of the variance attributable to the different levels of PA among the groups, indicating a strong relationship between PA levels and the tendency toward exercise dependence. The findings of this study showed the influence of the type of sport on BD, where football players and volleyball players had differences in BD, highlighting that beyond sex, the different physique demands of a sport could influence the perception of one’s body.

PA participation is associated with a multitude of positive outcomes, both physical (e.g., enhanced physical fitness through reduced body fat and increased muscle mass) and psychological (e.g., improved mood and self-esteem, alongside decreased anxiety and depression), which can contribute to a more positive body image [7,11,21,22,52]. The literature has mainly focused on the benefits of exercise interventions (strength vs. aerobic) or levels of PA commitment on body image [7,11,33,53,54]. However, in the present study, we also took into consideration the sedentary lifestyle (long sitting time), considering the evidence on the importance of the deleterious health consequences of prolonged sitting, which may be independent of the protective effect of regular PA [44]. In line with the literature [7,8,21], the present study confirms the central (positive) role of PA, particularly when it meets or exceeds public health recommendations, in individual body perceptions. In fact, the physically active men reported a more favorable body image than sedentary women, with the large ES indicating a 15% difference between groups. Although the sedentary women reached the minimum recommended amount to be classified as minimally active [36], the long time spent sitting could have influenced their BD. Investigating the differences between perceived and actual weight changes among university students during the COVID-19 pandemic, Keel et al. [55] reported that participants had a tendency to feel they had gained weight and were eating more, spending more time watching TV/movies and on social media, and gaming, although no significant changes in weight were reported. Despite the benefits of PA on both physical and psychological health, the deleterious impacts of prolonged sitting may attenuate these advantages, suggesting that focusing on reducing sitting time, alongside increasing PA levels, may be used as a health promotion strategy to reduce BD. Moreover, individuals with long sitting time could spend more time watching TV or on social media, which broadcast thinness ideals that are difficult to achieve without constant commitment to training and nutrition, fostering BD [56]. The significant differences in BD between the physically active men and the sedentary women, as opposed to the nonsignificant differences observed between the physically active men and sedentary men, underscore the combined influence of sex and the role of PA on individual body perceptions. This finding aligns with that of Fischetti et al. [57], who investigated the impact of sex and exercise differences on BD, reporting lower BD in physically active men than in inactive women, though the differences were not significant when comparing active men to their inactive counterparts. Furthermore, the differences observed between football and volleyball players provide additional evidence supporting the strong influence of sex on body image, highlighting that sex is a factor influencing individual BD. It is widely acknowledged that women tend to exhibit greater BD than their male counterparts [10,19,58,59,60]. According to Fredrickson and Roberts’ objectification theory [61], women are more likely to internalize an observer’s perspective as a primary view of their physical appeal, which may contribute to their increased BD. Therefore, these sex-based differences in perception might overshadow the positive changes brought about by PA and short sitting time. In fact, while PA can improve body image, the impact of sex-related social factors can be more influential, particularly in inactive populations [19]. Sex differences and engagement in physical training appear to play a role in shaping CBD. The sedentary women reported more positive values than the sedentary men, with the large ES indicating that 60.3% of the two groups overlapped, suggesting that women with long sitting time may perceive their body image as being less aligned with social norms than their male counterparts. The potential impact of sex on body image in athletes represent a topic where it is difficult to draw conclusions, given the contradictory findings in the literature. Francisco et al. [62] reported that gymnasts and ballet dancers, regardless of their sex, felt the pressure to be thin, while others [63,64,65] found that female athletes felt more pressured to fit a lean ideal and experienced more BD and a less positive body image. Investigating the relationship between intensive sporting practice and body dysmorphism, Iacolino et al. [66] reported that being female and having a higher level of difficulty in identifying feelings were predictive of the general level of body uneasiness, avoidance, and concerns about their body. In the present study, although football and volleyball players demonstrated body images differing from those of their less active peers, higher BD was found in volleyball players. Moreover, the large dimension of the ES indicated that 83.9% of the volleyball players’ group had a mean above the mean of the football players’ group, which, as suggested in the literature [15,67], could be attributed to differences in both sex and the type of sport. Indeed, sports can be classified as “aesthetic/lean” and “nonaesthetic/non-lean” [68], where BD seems to be higher in weight-sensitive (i.e., aesthetic) athletes, such as ballet dancers, who seem to be more dissatisfied than others due to the perception of being overweight with a greater desire to be thin, especially in female dancers. Although volleyball does not fall into this category, their training, focusing on upper limb strength [69], increases muscle mass in these areas, which may result in physiques differing from traditional female aesthetic ideals. Football training requires extensive aerobic and anaerobic work [70], developing physiques more aligned with society’s ideals, which could positively influence football players body image. The benefits of football practices on body image have been documented in the literature [71,72] investigating the effects of a football training program on body composition and body image satisfaction among preadolescents, reporting improvements in body composition and decreases in BD, suggesting the positive benefits of this sport on physical and psychological health. Therefore, given the physical demands of volleyball, players might experience more BD if they do not identify with their ideal body type. According to Steinfeldt et al. [73], volleyball players experience a paradox where women appreciate the power and strength of their bodies and acknowledge the importance of being muscular, although being aware of the contrasting societal body type expectations that contribute to their desire to avoid being perceived as too muscular and not conforming to traditional aesthetics norms of femininity. Moreover, collegiate women volleyball players internalized a physique as more muscular and athletic than nonathletes as not fitting with society’s ideals [74]. That study’s findings are in line with the present findings, where the volleyball players had higher values than those reported in the physically active men, where there was a 94.9% chance that a person picked at random from the volleyball players’ group had a higher score than a person picked from the physically active men. This difference further underscores the specific training completed by athletes in sports with highly specialized physical requirements in comparison with individuals taking advantage of the health benefits of exercise without a performance context. Although the involvement in sport “protected” athletes from body image concerns, this protection was less present in women. Therefore, given the interaction with sex [15,68,75], the effect of sport type on BD differed between men and women. The literature identifies a threshold value of higher than 30% in the BDI as indicative of a risk of body image disorders [49]. In the present study, no significant group differences were observed, and the threshold was not reached or exceeded. These findings could be attributed to the specific characteristics of the sample such as the participants’ body composition, as the participants predominantly presented a normal BMI, thus probably limiting the risk of body image disorders. Therefore, future research should consider incorporating a more diverse sample by including overweight and underweight populations to possibly provide more information into the relationship between PA commitment and body image disorders in a nonhomogeneous sample.

Although regular PA, exercise, and sports participation at various competitive levels are important for improving and maintaining mental and physical health, increasing the amount of physical training could lead to compulsive behaviors or addiction [23,76,77,78]. Our findings confirm that the prevalence of exercise addiction risk is generally higher among regular exercisers than in the general population [79,80]. In fact, the sedentary women had lower scores in the different dimensions of exercise dependence than the physically active men and volleyball players, highlighting the paradoxical phenomenon where long sitting time, despite the general health risks associated, could have a protective role on exercise behavior. However, these dissimilarities were not found in the sedentary men, highlighting sex differences in exercise dependence. The literature [25,26,27] suggests that men generally score higher in exercise dependence than women, probably due to social dynamics, where men have greater motivation to exercise and increase the amount and intensity of training, independent of their sitting time. It might be possible that, for men, exercise is essential for obtaining a strong and muscular physique, whereas women may find that exercise may not yield their desired (thin) physique [26], which is not achieved unless through caloric restriction in the dietary regimen.

Volleyball players had higher values for lack of control, time, reductions in other activities, tolerance, continuance, and EDS score than the sedentary women. This highlights the potential negative impact of sports, especially in continuance, where the large ES indicated a 92% likelihood that a randomly selected volleyball player would score higher than a randomly selected sedentary woman. Competitive athletes tend to exhibit more symptoms of exercise dependence than noncompetitive athletes. In fact, Condello et al. [21] reported that senior athletes showed significantly higher values in all dimensions of the EDS-21 than sedentary counterparts, indicating how sport commitment represents a risk of maladaptive exercise behaviors similar to those found in younger athletes. Due to their competitive nature and rigorous training demands, sports can lead athletes to push their limits to improve performance, which can result in an obsessive and compulsive relationship with their sport [21,23,76,79,81]. Although the volleyball players demonstrated differences from the sedentary women in several dimensions of the EDS, in line with the literature [82], these dissimilarities were not present in the football players, where individual-sport athletes had a higher risk of exercise dependency than team-sport ones. A systematic review [79] showed that distinct factors could play roles in the development of exercise dependence in sport practices, such as obsessive passion and dedication, social physique anxiety, eating disorders, and weight and shape concerns [79]. Weight concerns and BD could be relevant factors explaining the higher scores observed in several dimensions of exercise dependence in the volleyball players’ group. This might suggest an association between BD and exercise dependence, where individuals with negative perceptions of their body image may choose to excessively exercise as a method to enhance their physical appearance and achieve their aesthetic ideals [33,60]. A positive correlation was found [83] between thin-ideal internalization and compulsive exercise in college students, who increased their training to achieve their ideal body. Thin-ideal internalization also mediated the relationship between personality traits—such as neuroticism, extraversion, and conscientiousness—and exercise behaviors [83]. Consequently, BD is an important factor to consider in the exercise context, and understanding these dynamics is essential for developing more holistic approaches to managing exercise dependence, particularly in competitive sports settings [33].

The present study identified significant findings concerning body image and exercise dependence across various groups. However, the representativeness and the generalizability of these findings may be limited due to the characteristics and the type of sport included. Different sports require, among others, specific body compositions to achieve optimal performance [68]. Moreover, we considered the team sports most commonly practiced in Italy for men and women, although other types of sports should be targeted in future research. Sports like bodybuilding emphasize the need for high muscle hypertrophy and a low percentage of body fat, while dancers and gymnasts tend to require a thin and lean body [15,68]. Similarly, studies in sports like powerlifting and sumo, where athletes can gain performance-related benefits from increased body mass and fat [84,85], could enrich the theoretical assumptions made in the present study. Another limitation is that we considered different sports for each sex (football for men and volleyball for women). This approach was intentional for our study design, allowing us to have groups that were representative of the typical sports played in Italy. However, this choice could be a potential confounding factor, as the observed differences in body image and exercise dependence may have been influenced not only by the type of sport but also by sex. Therefore, future studies could investigate the effect of different sport practices including both sexes within each sport type to provide a clearer understanding of the impact of sport type on psychological outcomes. Lastly, although MANOVA was used in our analysis to investigate the effects of sitting time on BD and exercise dependence, it could also be interesting to explore the correlation between sitting time and these psychological outcomes, providing further insights into how sedentary behavior impacts body image and exercise dependence.

## 5. Conclusions

This study aimed to evaluate the effects of team sport participation, PA engagement level, and sitting time on body image and exercise behaviors according to sex. The results showed differences between the groups in BD, as well as higher levels of exercise dependence in physically active men and volleyball players with respect to women with longer sitting time. Specifically, this study found that volleyball players exhibited higher levels of BD and a greater risk of developing maladaptive exercise behaviors than football players and physically active men. These findings are in line with the hypothesis of this study that different lifestyles, such as practicing sport, engaging in PA, and long sitting time could differently influence the perception of one own’s body, also highlighting the importance of differences sex and the type of sport.

While PA and engagement in sports positively contribute to body image perception, particularly in men, excessive exercise can reduce these health benefits, especially in women. Furthermore, this study highlights the impact of sedentary behaviors on these outcomes, with participants sitting for long time showing differences in body image and exercise dependence compared to the other groups. Therefore, prolonged sitting could have a negative influence on body image and a protective role on reducing exercise behaviors, despite its general association with negative health effects. Thus, a careful balance between exercise and mental health is essential, particularly within competitive sports contexts. Achieving such a balance can help with reducing the risks of excessive dependency on exercise and having a healthy approach toward PA and sports. This research provides more insights into personalizing approaches for promoting healthy exercise habits across different populations and sport disciplines. By understanding these findings, it will be possible to develop interventions that balance PA and mental health, particularly in competitive sports contexts.

## Figures and Tables

**Table 1:** Means and standard deviations of the participants’ anthropometric characteristics.

	FPG	VPG	MPAG	FPAG	MSG	FSG
Age (years)	22.0 ± 3.4	22.0 ± 2.3	22.5 ± 3.5	22.4 ± 2.1	23.7 ± 1.9	23.5 ± 2.7
Body mass (kg)	67.3 ± 8.3	63.6 ± 7.7	73.5 ± 8.5	58.6 ± 7.5	76.8 ± 13.3	64.7 ± 8.2
Body height (cm)	171.9 ± 8.0	167.5 ± 7.2	175.9 ± 4.5	164.6 ± 4.4	178.1 ± 10.0	165.1 ± 6.1
BMI (kg·m^−2^)	22.7 ± 2.5	22.6 ± 2.2	23.7 ± 2.0	21.6 ± 2.7	24 ± 2.1	23.7 ± 2.8

BMI = body mass index; FPG = football players’ group, VPG = volleyball players’ group; MPAG = physically active men’s group; FPAG = physically active women’s group; MSG = sedentary men’s group; FSG = sedentary women’s group.

**Table 2:** Means and standard deviations of the IPAQ scores.

	FPG	VPG	MPAG	FPAG	MSG	FSG
Sitting (hour/day)	2.6 ± 1.7	3.0 ± 2.0	1.9 ± 0.6	2.1 ± 0.8	6.5 ± 1.7	7.0 ± 2.5
W-MET (min/week)	639.6 ± 771.0	556.2 ± 340.4	970.5 ± 1179.5	1244.1 ± 1162.0	925.8 ± 1368.3	1565.5 ± 1131.1
M-MET (min/week)	763.5 ± 858.4	1322.8 ± 911.9	824.7 ± 902.3	920.0 ± 782.5	476.2 ± 408.9	211.7 ± 344.0
V-MET (min/week)	1736.4 ± 1523.8	2800.0 ± 1927.9	3971.7 ± 1331.2	4032.0 ± 2911.5	1545.0 ± 1463.1	943.5 ± 1107.1
T-MET (min/week)	3139.6 ± 2409.3	4679.1 ± 2472.5	5767.0 ± 2346.4	6196.1 ± 3517.9	2947.1 ± 1597.9	2720.8 ± 1563.0

W-MET = walking metabolic equivalent of task; M-MET = moderate metabolic equivalent of task; V-MET = vigorous metabolic equivalent of task; T-MET = total metabolic equivalent of task; FPG = football players’ group, VPG = volleyball players’ group; MPAG = physically active men’s group; FPAG = physically active women’s group; MSG = sedentary men’s group; FSG = sedentary women’s group.

**Table 3:** Means and standard deviations of body image indices.

	FPG	VPG	MPAG	FPAG	MSG	FSG
BD (%)	1.2 ± 19.4	16.4 ± 9.4	−3.4 ± 7.6	4.5 ± 9.8	5.7 ± 13.5	11.6 ± 10.1
SxBD (%)	−0.2 ± 21.0	11.9 ± 15.3	0.2 ± 11.3	2.9 ± 14.6	7.5 ± 16.4	9.1 ± 12.4
CBD (%)	−14.3 ± 11.7	7.9 ± 14.3	3.1 ± 18.7	3.1 ± 14.2	−4.7 ± 17.4	16.1 ± 22.4
BDI (%)	14.9 ± 6.1	14.2 ± 9.4	10.5 ± 5.1	10.3 ± 5.7	12.7 ± 6.7	15.2 ± 8.3

BD = body dissatisfaction; SxBD = sexual body dissatisfaction; CBD = comparative body dissatisfaction; BDI = Body Dissatisfaction Index; FPG = football players’ group, VPG = volleyball players’ group; MPAG = physically active men’s group; FPAG = physically active women’s group; MSG = sedentary men’s group; FSG = sedentary women’s group.

**Table 4:** Means and standard deviations of exercise dependence values.

EDS-21 Dimension (AU)	FPG	VPG	MPAG	FPAG	MSG	FSG
Withdrawal Effects	8.9 ± 4.5	9.8 ± 4.5	7.7 ± 3.1	8.7 ± 3.5	8.6 ± 4.9	7.2 ± 4.2
Continuance	4.9 ± 3.7	12.7 ± 4.8	5.1 ± 2.1	4.2 ± 1.6	7.3 ± 3.7	4.8 ± 2.7
Tolerance	11.9 ± 3.7	12.7 ± 3.8	12.8 ± 2.7	11.8 ± 3.9	11.2 ± 4.5	7.8 ± 4.5
Lack of Control	7.4 ± 3.2	9.3 ± 4.2	8.3 ± 3.7	7.6 ± 2.8	6.7 ± 3.4	4.3 ± 2.0
Reduction in Other Activities	6.5 ± 2.2	8.4 ± 4.3	7.0 ± 3.4	5.2 ± 2.0	6.4 ± 1.9	4.7 ± 2.0
Time	11.2 ± 3.0	11.8 ± 3.7	11.7 ± 2.9	11.1 ± 3.4	9.3 ± 3.3	6.1 ± 3.4
Intention Effects	8.3 ± 2.8	9.2 ± 5.0	8.0 ± 3.1	8.0 ± 2.4	7.5 ± 4.0	5.3 ± 3.4
EDS score	59.2 ± 14.2	74.2 ± 20.4	60.7 ± 11.9	56.6 ± 14.2	57.31 ± 19.9	40.5 ± 18.8

EDS = Exercise Dependence Scale; FPG = football players’ group, VPG = volleyball players’ group; MPAG = physically active men’s group; FPAG = physically active women’s group; MSG = sedentary men’s group; FSG = sedentary women’s group.

## Data Availability

The data acquired and analyzed in the present study are available from the corresponding author upon reasonable request.
